# Factors Associated with a Lack of Willingness to Vaccinate against COVID-19 in Poland: A 2021 Nationwide Cross-Sectional Survey

**DOI:** 10.3390/vaccines9091000

**Published:** 2021-09-08

**Authors:** Filip Raciborski, Piotr Samel-Kowalik, Mariusz Gujski, Jarosław Pinkas, Magdalena Arcimowicz, Mateusz Jankowski

**Affiliations:** 1 Department of Prevention of Environmental Hazards and Allergology, Medical University of Warsaw, 02-091 Warsaw, Poland; filip.raciborski@wum.edu.pl (F.R.); piotr.samel@wum.edu.pl (P.S.-K.); 2 Department of Public Health, Medical University of Warsaw, 02-097 Warsaw, Poland; 3 Centre of Postgraduate Medical Education, School of Public Health, 01-826 Warsaw, Poland; jpinkas@cmkp.edu.pl (J.P.); mjankowski@cmkp.edu.pl (M.J.); 4 Department of Otorhinolaryngology, Head and Neck Surgery, Medical University of Warsaw, 02-097 Warsaw, Poland; magdalena.arcimowicz@wum.edu.pl

**Keywords:** COVID-19, SARS-CoV-2, vaccinations, vaccine hesitancy, vaccines, Poland

## Abstract

We aimed to assess the factors associated with a lack of willingness to vaccinate against COVID-19 among adults in Poland three months after the introduction of mass vaccination against COVID-19 in Poland. This cross-sectional study was carried out between 8 and 18 April 2021 on a representative nationwide sample of 1131 inhabitants of Poland aged 18 and over. Almost one-third of adult inhabitants of Poland (30%; 95%CI: 27.4–32.7%) declared a lack of willingness to vaccinate against COVID-19. Females had higher odds of refusing COVID-19 vaccination compared with males (OR = 1.68; 95%CI: 1.25–2.27). The lack of higher education was significantly (*p* < 0.001) associated with greater odds of refusing the COVID-19 vaccination. Participants living in rural areas compared with those living in the largest cities (over 500,000 inhabitants) had three times higher odds of refusing the COVID-19 vaccination (OR = 3.20; 95%CI: 1.71–6.01). Respondents who declared willingness to vote for one of the right-wing political parties publicly supporting the anti-vaccination movement in Poland had eight times higher odds (OR = 8.01; 95%CI: 3.65–17.60) of refusing the COVID-19 vaccination compared with other groups. Moreover, those who had three children or more, respondents who declared passivity towards participating in religious practices as well as active internet users had significantly higher odds of refusing the COVID-19 vaccination.

## 1. Introduction

On 21 December 2020, the European Medicines Agency (EMA) announced their decision on the conditional marketing authorization for the first coronavirus disease 2019 (COVID-19) vaccine in the European Union (EU) [1]. This decision allowed the implementation of vaccination programs against COVID-19 in the EU Member States. According to the EU Vaccines Strategy, the EU Member States launched the joint purchase of COVID-19 vaccines to secure supplies and facilitate the rollout of vaccines [2]. The European Commission has signed the Advance Purchase Agreements with individual COVID-19 vaccine producers on behalf of Member States, wherein the volume of orders was proportional to the number of inhabitants of individual EU Member States [3]. According to the EU Vaccines Strategy, by summer 2021, Member States should have vaccinated a minimum of 70% of the entire adult population, in order to achieve herd immunity [2,3,4]. While vaccine deliveries depended on the EU Agreements, the organization of COVID-19 vaccination programs, methods of promotion as well as priority groups for COVID-19 vaccination differed between EU Member States [2,4].

Poland is the fifth most populous EU country. The public authorities had ordered 60 million doses of the COVID-19 vaccine from six suppliers [4]. To organize mass vaccination against COVID-19 in Poland, the National COVID-19 Vaccination Program was developed. The Program specified the rules of vaccination and places in which the vaccination was to take place as well as the order of vaccination of individual subpopulations [4]. The rules of the Program have changed with the new EMA recommendations as well as the recommendations of the national public health authorities [1,5]. In Poland, the healthcare and social workers were among the first to be vaccinated against COVID-19 in Poland. On 27 December 2020, the first Polish citizen—a nurse from the largest COVID-19-dedicated hospital—was vaccinated against COVID-19 [6]. In January 2021, an age-based program was applied. Vaccination of older adults began on 25 January 2021, with priority for registration for people over 80 years of age. In the following weeks, individual age groups (by year of birth) had an opportunity to register for COVID-19 vaccination. On 10 May 2021, all Polish inhabitants aged 18 and over could register for a COVID-19 vaccination [4,5].

Vaccination against COVID-19 is voluntary throughout the European Union. From 1 July, the EU Digital COVID Certificate (that covers vaccination, test or recovery) is in force in EU member states [5]. The Certificate facilitates the free and safe circulation of citizens in the EU during the COVID-19 pandemic as well as allowing access to selected facilities and services. Activities undertaken by national education and various information campaigns in order to promote COVID-19 vaccination have not shown the desired results. A significant proportion of EU citizens declares a lack of willingness to be vaccinated despite facilitating the circulation of citizens in the EU.it.

The introduction of the COVID-19 vaccinations meant that every adult must decide whether to vaccinate against COVID-19. Until now, public debate on adult immunization has been limited to the influenza vaccination. In Poland, influenza vaccination coverage rates do not exceed 5% of the population, as opposed to the Netherlands or the United Kingdom, where the influenza vaccination coverage rates exceed 60% of the population [7,8]. Moreover, in Poland, there is a formalized and structured anti-vaccination movement, which tries to abolish mandatory childhood vaccination. During the COVID-19 pandemic, the anti-vaccination movement also questioned the importance and safety of vaccination against COVID-19 [9,10]. Identification of the factors associated with negative attitudes towards the COVID-19 vaccine will allow for the implementation of educational activities that build confidence in vaccination. Moreover, these data may be the basis for identifying best practices in the promotion of vaccination against COVID-19 in EU countries, with particular emphasis on the Central and Eastern Europe.

We aimed to assess the factors associated with a lack of willingness to vaccinate against COVID-19 among adults in Poland three months after the introduction of mass vaccination against COVID-19 in Poland.

## 2. Materials and Methods

### 2.1. Study Design

This cross-sectional study was carried out between 8th and 18th of April 2021 on a representative nationwide sample of 1131 individuals aged 18+ in Poland. Computer-assisted personal interviewing (CAPI), computer-assisted telephone interviewing (CATI) and computer-assisted web interviewing (CAWI) were used. Each respondent could choose the preferred method of survey.

The surveying process had been carried out by a specialized survey company—Public Opinion Research Center (Warsaw, Poland). The Public Opinion Research Center (CBOS) conducts a national cross-sectional survey on the population’s opinion regarding political and social issues in Poland [11].

### 2.2. Population

The respondents were adult inhabitants of Poland aged 18–90 years, randomly selected from the PESEL register (the national register in Poland of inhabitants). The PESEL database is an official database managed by the Ministry of Internal Affairs and Administration. The sampling frame was based on demographic data included in PESEL register (data on the number of registered residents in all localities of the country, age and gender). The results are weighted to the data of Statistics Poland (Central Statistical Office) and demographic data from the PESEL register. The following characteristics of the respondents were used for weighing: gender, city/village class, voivodeship of residence, age group, education level, social and professional group. In total, 1131 individuals were included in this study.

### 2.3. Measures

The study was conducted in April 2021. Questions concerning the COVID-19 pandemic, perception of anti-epidemic measures implemented by public authorities, attitudes towards the COVID-19 vaccine, and the willingness to be vaccinated had been included in the questionnaire. The questions (Supplementary Figure S1) also addressed personal characteristics, including age, sex, size of the place of residence, education, political preferences, religious faith and practice (without specifying a particular religion) and internet use.

Willingness to vaccinate against COVID-19 was defined using the question: “Would you like to be vaccinated against COVID 19?”. Respondents who indicated negative attitudes towards COVID-19 vaccination by choosing the answers: “Definitely not” or “Rather not” were classified as those who declare lack of willingness to vaccinate against COVID-19.

For the needs of the logistic regression model, quantitative variables describing age was divided into 5 categories and then recoded into series 0–1 variables (dummy variables). In the same way, the variable describing the number of children under 18 was transformed, finally creating a series of four 0–1 variables.

When asked about participation in religious practices, 34 individuals refused to answer. For analytical purposes, these participants were added to those who declared that they did not participate in religious practices. Moreover, 9 individuals refused to answer when asked about using the Internet. For the purposes of these analyses, these participants were added to those who declared that they did not use the Internet.

In terms of political preferences, the willingness to vote for a candidate of one of the right-wing political parties was analyzed. This right-wing political party is the only political party in the Polish Parliament (Sejm) that publicly supports the anti-vaccination movement in Poland as well as denying the importance of vaccination against COVID-19 and the threat posed by COVID-19 [12].

### 2.4. Statistical Analysis

The data were analyzed with SPSS V.27 (Armonk, NY, USA: IBM Corp). Demographic weighting was applied. A multivariate logistic regression model, crosstabs and descriptive statistics were used for statistical analysis. The chi-squared test was performed for bivariate analyses. The level of statistical significance was set at 0.05.

### 2.5. Ethics

This study was carried out following the principles expressed in the Declaration of Helsinki. This study is a secondary statistical analysis. Data used in this study were collected by a specialized survey company—Public Opinion Research Center (CBOS). Datasets were anonymous and prevented the identification of any individual study subject by the research team at any stage of the study.

## 3. Results

### 3.1. Characteristics of the Study Population

The analysis is based on responses received from 1131 individuals (52.9% females). Table 1 shows the demographic characteristics of the study sample. The mean age was 48.2 (±17.8) years. Among participants, 31.6% had secondary education and 28.6% had higher education. Most of the respondents (59.4%) lived in cities. Almost three-quarters of the respondents used the Internet (Table 1).

### 3.2. Percentage of Respondents, Who Declared a Lack of Willingness to Vaccinate against COVID-19 by Sociodemographic Factors

Almost one-third of adult inhabitants of Poland (30%; 95%CI: 27.4–32.7%) declared a lack of willingness to vaccinate against COVID-19. The highest percentage of respondents, who declared lack of willingness to vaccinate against COVID-19 (72.3%; 95% CI: 57.5–84.4%) was observed in the group of people who declared support for one of the right-wing political parties. Negative attitudes towards the COVID-19 vaccine were also relatively frequent in the group of people with three or more children—(51%; 95% CI: 38.2–65.7%). Moreover, almost half of respondents aged 18–29 years (45.9%; 95% CI: 38.8–52.7%) declared lack of willingness to vaccinate against COVID-19. The lowest percentage of respondents who declared a lack of willingness to vaccinate against COVID-19 was observed among those aged 60–74 years (14.8%; 95% CI: 11.1–19.5%) as well as inhabitants of the largest cities above 500,000 residents (13.5%; 95% CI: 8.0–20.6%). Details are presented in Table 2.

Of the 491 respondents who have not yet been vaccinated but declared their willingness to vaccinate against COVID-19, 60.7% (95% CI: 56.3–64.9%) indicated that they care about which COVID-19 vaccine they will receive. In this group, the COVID-19 vaccine from Pfizer-BioNTech (70.1%) was the most preferred. The Moderna COVID-19 vaccine was preferred by 27.9%, Johnson & Johnson’s single-dose COVID-19 vaccine was indicated by 16.1% and AstraZeneca was preferred by 6.7% of respondents.

### 3.3. Factors Associated with the Intention to Not Pursue COVID-19 Vaccination

The multivariate logistic regression that explained the lack of willingness to vaccinate against COVID-19 obtained Cox and Snell R-Squared of 0.147 and Nagelkerke R-Squared of 0.209. Respondents who declared willingness to vote for one of the right-wing political parties (political party publicly supporting the anti-vaccination movement in Poland) had eight times higher odds (OR = 8.01; 95%CI: 3.65–17.60) of refusing the COVID-19 vaccination compared with respondents who support other political parties or do not intend to participate in the elections. Participants living in rural areas had three times higher odds of refusing the COVID-19 vaccination (OR = 3.20; 95% CI: 1.71–6.01) compared with those living in the largest cities (over 500,000 inhabitants). Lack of higher education was significantly (*p* < 0.001) associated with greater odds of a lack of willingness to vaccinate against COVID-19. When compared with those aged 75 and over, participants aged 60–74 years had more than three times lower odds of refusing the COVID-19 vaccination (OR = 0.30; 95% CI: 0.16–0.58). Females had higher odds of refusing the COVID-19 vaccination compared with males (OR = 1.68; 95% CI: 1.25–2.27). Moreover, having three children or more, passivity towards participating in religious practices as well as being an internet user were significantly associated with a lack of willingness to vaccinate against COVID-19. Details are presented in Table 3.

## 4. Discussion

To the best of the author’s knowledge, this is the most up-to-date study on attitudes towards COVID-19 vaccines in Poland, carried out three months after the introduction of mass vaccination against COVID-19 in Poland. This study showed that 70% of adults in Poland are willing to get vaccinated against COVID-19. However, females, respondents without higher education, those living in rural areas, respondents who declared willingness to vote for one of the right-wing political parties, those who had three children or more, respondents who declared passivity towards participation in religious practices as well as active internet users had significantly higher odds of refusing the COVID-19 vaccination.

In April 2021, Common Vaccination Centers, large facilities organized in central places of towns and villages (some even at sports stadiums), started operating; therefore, every inhabitant of Poland had the opportunity to get vaccinated against COVID-19 in the vicinity of their place of residence [4,6]. The opening of the Common Vaccination Centers was preceded by a wide media campaign encouraging COVID-19 vaccination. Because of the widespread access to Vaccination Points (more than 7600 facilities across the country) and the dissemination of information on how to sign up for vaccination, it seems that our study presents the most up-to-date data on factors associated with attitudes towards the COVID-19 vaccination in Poland.

Out of eight predictors of the lack of willingness to vaccinate against COVID-19, respondents who declared willingness to vote for one of the right-wing political parties had eight times higher odds of refusing the COVID-19 vaccination compared with respondents who support other political groups or do not intend to participate in the elections. This political party is the only political party in the Polish Parliament (Sejm) that publicly supports the anti-vaccination movement in Poland [12]. The leaders of the anti-vaccine movement took part in the 2019 parliamentary elections with the support of this right-wing political party (joint slate) [9,12]. Moreover, political leaders of this political party publicly denied the importance of anti-epidemic measures to mitigate the spread of SARS-CoV-2 infections and presented negative attitudes towards vaccination against COVID-19, which may cause vaccine hesitancy among inhabitants of Poland. Our analyses did not reveal any associations between the support of other political parties and the negative attitudes towards COVID-19 vaccines. Our findings confirm the significance of the impact that politicians’ statements have on social attitudes towards vaccination against COVID-19.

This study showed that living in rural areas or cities below 100,000 inhabitants was associated with higher odds of refusing the COVID-19 vaccination. This observation may result from the fact that due to the population density, level of urbanization and industrialization, the number of COVID-19 cases and COVID-19-related deaths reported in rural areas and smaller towns was lower compared with large urban centers [13,14]. Due to the differences in the dynamics of COVID-19 pandemic between rural and urban areas, the percentage of respondents who declare fear of COVID-19 and subsequent willingness to vaccinate against COVID-19 among those who lived in these areas may be lower than that among inhabitants of large cities, where the risk of SARS-CoV-2 infection (e.g., in public transport or in a shop) seems to be higher [15].

Currently, there are no conclusive data on the impact of an individual’s educational level on their COVID-19 vaccine trust [16,17,18]. An analysis of the relationship between COVID-19 and age, gender and level of education showed that the impact of education level on COVID-19 vaccine trust differed across countries. The analysis was conducted in June of 2020 and included 13,426 individuals from 19 high-COVID-19 burden countries [16]. Highly educated individuals in Ecuador, France, Germany, India, and the US reported that they will accept a COVID-19 vaccine, contrary to Canada, Spain and the UK, where the higher education levels were associated with lower COVID-19 vaccination acceptance [16]. In this study, the lack of higher education was significantly associated with less willingness to vaccinate against COVID-19. This observation is contrary to studies on factors associated with attitudes towards compulsory childhood vaccinations in Poland, where the educational level did not influence attitudes towards vaccinations [9,10].

Numerous studies showed that females are more hesitant to get vaccinated against COVID-19 [19,20,21]. Additionally, in this study females had higher odds of refusing the COVID-19 vaccination compared with males. However, our observation is contrary to a previous study from Poland (Babicki and Mastalerz-Migas) [22], where the women demonstrated more favorable attitudes toward getting vaccinated against COVID-19 [22]. The differences between our study and the study by Babicki and Mastalerz-Migas may also result from different methods of data collection as well as different study periods.

The impact of age on attitudes towards COVID-19 vaccines was observed only in the case of participants aged 60–74 years, who had more than three times lower odds of refusing the COVID-19 vaccination compared with those aged 75 years and over. This observation is in line with other studies, where older people (e.g., in Canada, France, Germany, Sweden) were significantly more prone to vaccination than younger groups [16].

In this study, respondents with at least three children had significantly higher odds of refusing the COVID-19 vaccination compared with those without children. We can hypothesize that people with more than two children are more likely to receive anti-vaccine content as they repeatedly decide whether to vaccinate their children, but this hypothesis requires further investigation [10].

Passivity towards participating in religious practices was another factor that significantly affected attitudes towards the COVID-19 vaccine. We can assume that the attitude towards religious practice reflects other social characteristics and behaviors, so it is not possible to unequivocally explain this observation. The positive stance of religious authorities on vaccination against COVID-19 may encourage participants of religious practice to vaccinate, especially among people over 60 years of age [23].

An infodemiological study showed that during the COVID-19 pandemic, the Internet was the major source of misinformation on COVID-19 [24,25]. Montagni et al. showed that the acceptance towards COVID-19 vaccines is associated with the ability to detect fake news and health literacy [26]. Internet users may be exposed to disinformation, especially on social media. The Internet and social media are the main communication channels for the anti-vaccination movement in Poland [9]. In this study, a lack of willingness to vaccinate against COVID-19 was associated with the use of the Internet. We can assume that these respondents were exposed to misinformation regarding COVID-19 on the Internet, which contributed to negative attitudes towards vaccination against COVID-19.

Our study showed that in April of 2021, 70% of adults in Poland are willing to get vaccinated. However, real-world data are needed in order to verify these declarations with social behaviors. As of 26 August 2021, cumulative uptake of at least one dose of the COVID-19 vaccine among adults in Poland amounts to around 59% [27], which is lower than declared by the respondents in our study. We can hypothesize, that misinformation about vaccinations, the presence of new variants of the virus, with reduced vaccine effectiveness, and the lack of fear of coronavirus, especially during the holiday season in Poland (June–August), resulted in lower uptake of the COVID-19 vaccine than declared in April, when the third wave of the COVID-19 pandemic peaked.

This study has practical implications for policymakers and public health authorities. First, the eight factors that were described in this study and are associated with attitudes towards COVID-19 vaccination can be a source of information on the target group, the persuasion of which to vaccinate will increase the chance of achieving herd immunity. Secondly, our research may be the basis for future research on health literacy among inhabitants of Poland. Moreover, the experience that results from the implementation of the National COVID-19 Vaccination Program can be used to plan communication towards building confidence in vaccination in Poland.

This study has several limitations. First, we used secondary data on attitudes towards COVID-19 vaccines, so the scope of the analysis is limited to available data. Secondly, this study was based on the results of a poll conducted in April 2021. Public opinion may change because of media campaigns and vaccination promotions by public authorities and medical professionals. Nevertheless, this is the most up-to-date study on attitudes towards COVID-19 vaccines in Poland, carried out three months after the introduction of mass vaccination against COVID-19 in Poland.

## 5. Conclusions

In April 2021 in Poland, females, respondents without higher education, those living in rural areas or cities below 100,000 inhabitants, respondents who declared willingness to vote for one of the right-wing political parties, those who had three children or more, respondents who declared passivity towards participating in religious practices as well as active internet users had significantly higher odds of refusing the COVID-19 vaccination.

Identifying groups that have negative attitudes towards the COVID-19 vaccination is crucial to developing personalized communication to build COVID-19 vaccine confidence.

## Figures and Tables

**Table 1:** Characteristics of the study group (*n* = 1131).

Variable	*n*	%
Overall	1131	100
Gender		
Female	598	52.9
Male	533	47.1
Age (years)		
18–29	197	17.4
30–44	328	29.0
45–59	250	22.1
60–74	281	24.8
75 and over	76	6.7
Educational level		
Primary	186	16.4
Vocational	265	23.4
Secondary	357	31.6
Higher	323	28.6
Place of residence		
Rural	459	40.6
City up to 99,000 residents	396	35.0
City between 100,000–499,000 residents	165	14.5
City above 500,000 residents	112	9.9
Occupational Status		
Active	620	54.8
Passive	511	45.2
Having children under 18 years of age		
No	752	66.5
Yes, one child	187	16.5
Yes, two children	144	12.7
Yes, three children or more	48	4.3
Participation in religious practices		
Yes, at least once a week	417	36.9
Yes, several times a year	384	34.0
Not at all	329	29.1
Willingness to vote for one of the right-wing political parties		
Yes	40	3.5
No	1091	96.5
Using the Internet		
Yes	840	74.2
No	291	25.8

**Table 2:** Percentage of respondents, who declared a lack of willingness to vaccinate against COVID-19 by sociodemographic variables, Poland, April 2021.

Variable	*n* *	% (95%CI)	*p*-Value
**Gender**			
Female	598	31.6 (28.0–35.4)	0.217
Male	533	28.2 (24.5–32.1)	
**Age (years)**			
18–29	197	45.9 (38.8–52.7)	<0.001
30–44	328	35.6 (30.6–41.0)	
45–59	250	26.4 (21.2–32.1)	
60–74	281	14.8 (11.1–19.5)	
75 and over	76	31.9 (22.0–42.6)	
**Educational level**			
Primary	186	33.5 (26.9–40.3)	<0.01
Vocational	265	31.1 (25.6–36.7)	
Secondary	357	34.6 (29.9–39.8)	
Higher	323	21.9 (17.7–26.7)	
**Place of residence**			
Rural	459	36.1 (31.9–40.6)	<0.001
City up to 99,000 residents	396	31.4 (26.9–36)	
City between 100,000–499,000 residents	165	20.5 (15.0–27.3)	
City above 500,000 residents	112	13.5 (8.0–20.6)	
**Occupational Status**			
Active	620	33.6 (29.9–37.3)	<0.01
Passive	511	25.6 (22.0–29.6)	
**Having children under 18 years of age**			
No	752	26.3 (23.3–29.6)	<0.001
Yes, one child	187	31.9 (25.7–39.0)	
Yes, two children	144	39.6 (31.9–47.7)	
Yes, three children or more	48	51 (38.2–65.7)	
**Participation in religious practices**			
Yes, at least once a week	417	25.2 (21.2–29.5)	<0.05
Yes, several times a year	384	34.1 (29.5–39.0)	
Not at all	329	31.2 (26.5–36.5)	
**Willingness to vote for one of the right-wing political parties**			
Yes	40	72.3 (57.5–84.4)	<0.001
No	1091	28.4 (25.8–31.1)	
**Using the Internet**			
Yes	840	33.1 (30.0–36.3)	<0.001
No	291	21.0 (16.6–25.9)	

* total number of respondents in the category.

**Table 3:** Sociodemographic factors associated with a lack of willingness to vaccinate against COVID-19—the multivariate logistic regression model.

Variable	*n*	*p*-Value	OR	95%CI OR	
				Lower	Upper
**Gender**					
Female	598	<0.001	1.68	1.25	2.27
Male	533	Ref. *	Ref.	Ref.	Ref.
**Age (years)**					
18–29	197	0.518	1.27	0.61	2.63
30–44	328	0.459	0.75	0.36	1.60
45–59	250	0.081	0.53	0.26	1.08
60–74	281	<0.001	0.30	0.16	0.58
75 and over	76	Ref.	Ref.	Ref.	Ref.
**Educational level**					
Primary	186	<0.001	3.12	1.84	5.27
Vocational	265	<0.001	3.10	1.96	4.89
Secondary	357	<0.001	2.17	1.48	3.18
Higher	323	Ref.	Ref.	Ref.	Ref.
**Place of residence**					
Rural	459	<0.001	3.20	1.71	6.01
City up to 99,000 residents	396	<0.001	2.93	1.56	5.50
City between 100,000–499,000 residents	165	0.46	1.31	0.65	2.65
City above 500,000 residents	112	Ref.	Ref.	Ref.	Ref.
**Occupational Status**					
Active	620	0.20	1.27	0.88	1.82
Passive	511	Ref.	Ref.	Ref.	Ref.
**Having children under 18 years of age**					
No	752	Ref.	Ref.	Ref.	Ref.
Yes, one child	187	0.58	0.89	0.59	1.34
Yes, two children	144	0.23	1.33	0.84	2.10
Yes, three children or more	48	<0.01	2.54	1.30	4.95
**Participation in religious practices**					
Yes, at least once a week	417	Ref.	Ref.	Ref.	Ref.
Yes, several times a year	384	0.05	1.40	1.00	1.97
Not at all	329	<0.05	1.50	1.04	2.16
**Willingness to vote for one of the right-wing political parties**					
Yes	40	<0.001	8.01	3.65	17.60
No	1091	Ref.	Ref.	Ref.	Ref.
**Using the Internet**					
Yes	840	<0.05	1.76	1.13	2.75
No	291	Ref.	Ref.	Ref.	Ref.

* reference category.

## Data Availability

The datasets generated during and/or analyzed during the current study are available from the corresponding author on reasonable request.

## Supplementary Materials

The following are available online at https://www.mdpi.com/article/10.3390/vaccines9091000/s1, Figure S1: Translated version of the study questionnaire.
