# Supplementary material for: Factors Associated with a Lack of Willingness to Vaccinate against COVID-19 in Poland: A 2021 Nationwide Cross-Sectional Survey
Source: Vaccines (Basel). 2021 Sep 8;9(9):1000. doi: 10.3390/vaccines9091000 (PMC8472927; doi:10.3390/vaccines9091000)
Supplement: Supplementary file 1 [file vaccines-09-01000-s001.zip › vaccines-1310864-supplementary.pdf]

Supplementary Figure S1. Translated version of the study questionnaire

Questions	Answers
**Year of birth of the respondent**	• Numeric
**Sex of the respondent**	• Male<br>• Female
**Place of residence**	• Please declare
**M1. Do you currently work for profit (full-time, in your own company or farm, or do you undertake commissioned work)?**	• Yes, part-time<br>• Yes, full time<br>• Yes, casual<br>• Not
**M4. What is your education? Please provide the highest level of education achieved by you.**	• Incomplete primary or no school education<br>• Primary<br>• Junior high school<br>• Basic Vocational (also SPR)<br>• Secondary general education without the matura exam<br>• Secondary general education with high school diploma<br>• Secondary vocational without high school diploma<br>• Secondary vocational with high school diploma (technical high school, vocational or technical high school)<br>• Post-secondary or post-secondary<br>• Higher with the title of engineer, bachelor, certified economist<br>• Higher with a master's degree, doctor or equivalent<br>• Higher with a doctoral degree or higher
**M5. How many people, including yourself, does your household consist of?**	• Numeric
**M6. How many adults, i.e. those aged 18 or over, make up your household? Please include yourself as well.**	• Numeric
**9. If the elections to the Sejm and Senate were held next Sunday, would you take part in them?**	• I would definitely take part in them<br>• I don't know if I would take part in them<br>• I certainly would not take part in them<br>• Refusal to answer

**10. Which party of the party would you vote for in the parliamentary elections?**	• Prawo i Sprawiedliwość (wraz z Solidarną Polską i Porozumieniem)<br>• Koalicja Obywatelska (Platforma Obywatelska, Nowoczesna, Inicjatywa Polska, Zieloni)<br>• Lewica (Nowa Lewica (dawniej SLD), Wiosna, Lewica Razem)<br>• Konfederacja Wolność i Niepodległość (KORWiN, Ruch Narodowy, Braun)<br>• Polskie Stronnictwo Ludowe - Koalicja Polska (Polskie Stronnictwo Ludowe, UED)<br>• Polska 2050 Szymona Hołowni<br>• Kukiz '15<br>• other party<br>• It's hard to say, I don't know yet, I hesitate<br>• Refusal to answer
**24. Are you personally afraid of coronavirus infection?**	• I am afraid<br>• Yes, I'm a little scared<br>• No, I'm not afraid<br>• No, I'm not afraid at all<br>• Hard to say<br>• Refusal to answer
**25. Would you like to get vaccinated against COVID-19?**	• Definitely yes<br>• Probably yes<br>• Probably not<br>• Definitely not<br>• I am already vaccinated<br>• Hard to say<br>• Refusal to answer
**26. Why would you not want to be vaccinated against COVID-19?**	• I am concerned about the side effects of the COVID-19 vaccine<br>• I am concerned that the COVID-19 vaccine will not be effective<br>• I avoid vaccinations at all<br>• I have already had COVID-19<br>• I believe that COVID-19 is not a serious disease<br>• Other reasons
**27. For what other reasons would you not want to be vaccinated against COVID-19?**	Text

**M7. Do you take part in religious practices such as: masses, services or religious meetings?**	• Yes, usually a couple of times a week<br>• Yes, once a week<br>• Yes, approximately once or twice a month<br>• Yes, several times a year<br>• I do not participate in them at all<br>• Refusal to answer
**M10. Do you use the Internet (websites, e-mail, instant messaging, etc.) at least once a week?**	• Yes<br>• No<br>• Refusal to answer
